# Supplementary material for: Exploring burn first aid knowledge and water lavage practices in Uganda: A cross-sectional study
Source: PLoS One. 2025 Feb 3;20(2):e0318087. doi: 10.1371/journal.pone.0318087 (PMC11790111; doi:10.1371/journal.pone.0318087)
Supplement: S1 Appendix — (DOCX) [file pone.0318087.s001.docx]

### QUESTIONNAIRE:

***For the questions 1 – 14 below, please chose one option or a fill with a short answer where needed***

1. Nationality
   - Ugandan
   - Non-Ugandan
2. Current age:.…………………….years
3. Please chose who you currently identify with:
   - Currently a burn victim
   - Care taker of a burn victim
   - None of the above
4. Gender:
   - Male
   - Female
5. Residency:
   - Urban
   - Rural
6. Level of education:
   - Less than primary
   - Primary
   - High school
   - College
   - University
   - Master’s degree
7. Current job:
   - Private work
   - Employed
   - Does not work
   - Other:…………….
8. Monthly earnings:
   - Between 50,000-100,000
   - Between 101,000-200,000
   - Between 201,000-500,000
   - Between 501,000-990,000
   - Between 1- 2 million
   - Above 2 million
   - Not applicable
9. Residence:
   - Central
   - Western
   - Eastern
   - Northern
   - ……………………………(others, please specify)
10. Number of children
    - 1
    - 2 - 3
    - >3
    - Not applicable
11. Do you any children under 18 living at home with you?
    - Yes
    - No
12. Have you have ever experienced a burn?
    - Yes
    - No
13. Have you ever cared for a person with a burn?
    - Yes
    - No
14. Have you ever received any information on Burn first Aid
    - Yes
    - No

If yes, what was the source of information? Choose all that apply.

- - Medical worker
  - Social media
  - Conferences
  - Workshops
  - Lectures
  - Friends and family
  - News
  - Radio
  - Internet websites
  - Newspaper
  - Television
  - Official first aid courses
  - Pamphlet
  - Health Campaigns

***Questions 15 – 22 are for Current Burn Victims ONLY:***

15. Site of Accident:

(a) At home
(b) School
(c)  Office/Workshop
(d)  On the Road
(e)  Others {Please specify]…………………………………….

16. Cause of burn

(a)  Fire from any source
(b)  Hot water/Scald
(c)  Electricity
(d)  Chemicals [please specify if the name is known]………………….

17. Was any First aid administered?

Yes

No

18. Who administered the First Aid

(a)  Parents
(b)  Relations/Guardians
(c)  Neighbor
(d)  Passerby
(e)  Others [please specify]

19. Was the First Aider educated

Yes
 No

Do not know

20. What was used for the First Aid ( you can choose more than one)

Water lavage
 Automotive Engine Oil/Brake fluid
 Herbal Preparations
 Silver Sulphadiazine
 Honey

Egg white

Animal fur

Aloe vera

Flour/salt/sugar

Mud

Animal fur

Toothpaste
 Gentian Violet
 Mud
 Human/Animal Urine
 Others [Please specify]……………………………………………………………………

21. If Water Lavage was used, please answer the following questions;

(a) What was the source of water – please specify:-

Well

Tap water

Bore hole

Others…………………………………………………………………………….

(b) What was the temperature of the water used – please specify:-

Ice at zero

Room temperature water

Warm water

(c) What was the approximate duration of the water lavage – please specify in minutes –

less than 5 minutes

6 – 10 minutes

11 – 15 minutes

16 – 20 minutes

21 – 30 minutes

more than 30 minutes

Do not know

22 (a) Time interval between onset of injury and arrival at the hospital
.………………………………………………………………………………………………

(b) Diagnosis at time of injury

……………………………………………………………………………………………………………………………………………………………………………………………………………………………………

**Prompted Questions 23 – 30 . For all participants.**

23 Burns can lead to permanent injuries .

Agree  Disagree

24.Burn injuries can lead to mental disorders.

Agree  Disagree

25.In case of burn injury, covering the burned area before heading to the hospital can decrease the risk of infection .  Agree  Disagree

26. In case of burn injury, picking blisters is an incorrect action

Agree  Disagree

27.Applying first aid medicine at home on a burned area leads to a better outcome

Agree  Disagree

28.In case of burn injury, it is beneficial to use antibiotics for management  Agree  Disagree

29In case of electrical burn injury, I should not touch the injured person if he/she is still in contact with the electrical current  Agree  Disagree

30.In case of electrical burn injury, the first action is to turn off the source of electricity if possible  Agree  Disagree

**Scenario Questions 31 a-d. For all participants.**

Would you please read through the following scenarios and record your immediate first aid management by selecting one of the options you would do in the first 10-20minutes? If you have any questions, please don’t hesitate to ask. Please answer all the questions. If you are unsure, please tick “Do not know” rather than leaving it blank.

a).A two-year-old boy dressed in a T-shirt and nappy reaches up to the stove and pulls a kettle of boiling water onto himself. What would you do?

1. Leave all clothing intact, wrap in a clean towel, seek help
2. Leave all clothing intact and cool with iced water
3. Remove all clothing and apply ice packs
4. Remove all clothing and cool with running water for 20 minutes
5. Do not know

b).A 10-year-old girl wanders barefoot into a burning bush as her family prepares the garden for crop cultivation. What do you do?

1. Immediately put out the fire, wrap in clean cloth /towel, seek help
2. Leave fire alone and cool with iced water
3. Transport to the nearest hospital and apply ice packs
4. Immediately access cool running water for 20 minutes
5. Do not know

c).A 25-year-old man is standing close to a candle at a party when his shirt catches fire. What should you do?

1. Leave all clothing intact, drop to the ground, and douse in cold water
2. Leave all clothing intact, instruct him to fall to the ground, and rollover
3. Remove all clothing, apply ice packs
4. Remove all clothing and cool with running water for 20 minutes
5. Do not know

d).A 30-year-old woman is cleaning her swimming pool, and some hydrochloric acid splashes onto her face. What would you do?

1. Leave all clothing intact, wrap in a clean towel
2. Leave all clothing intact, instruct her to jump into the swimming pool
3. Remove all clothing, apply ice packs
4. Remove all clothing and cool in shoer for 20 minutes
5. Do not know

**All participants.**

32a).Have you had a burn or scald in the last 12 months?

Yes
 No

b) If yes, what did you apply

Automotive Engine Oil/Brake fluid
 Herbal Preparations
 Silver Sulphadiazine

Water lavage
 Honey

Flour/salt/sugar

Toothpaste
 Gentian Violet
 Tomato paste

Animal fur

Aloe vera
 Human/Animal Urine
 Others [Please specify]……………………………………………………………………

………………………………………………………………………………………………………………………………………………………………………………………………………………………………………………………………………………………………………………………………………………………………………………………………………....

33. What is the recommended first aid treatment for a burn or scald?

Automotive Engine Oil/Brake fluid
 Herbal Preparations
 Silver Sulphadiazine

Water lavage
 Honey

Flour/salt/sugar

Toothpaste
 Gentian Violet
 Mud
 Human/Animal Urine
 Others [Please specify]……………………………………………………………………

………………………………………………………………………………………………………………………………………………………………………………………………………………………………………………………………………………………………………………………………………………………………………………………………………....

34When someone has a burn, it is recommended that you should apply cold running water. Do you know for how long you should apply cold running water?

less than 5 minutes

6 – 10 minutes

11 – 15 minutes

16 – 20 minutes

21 – 30 minutes

more than 30 minutes

Do not know

35Have you had first aid training in the last 12 months?

Yes……………*if yes, did it include burns component*…………………
 No

36How would you like to receive information for learning more about first aid for burn first aid?

- During hospital visit
- Social media
- Leaflets / posters
- Television advert
- Radio
- Internet websites
- Newspaper
- Telephone text message
- Mails
- Don’t know
- Others(please specify)…………………………………………………………………
