# Supplementary material for: Exploring burn first aid knowledge and water lavage practices in Uganda: A cross-sectional study
Source: PLoS One. 2025 Feb 3;20(2):e0318087. doi: 10.1371/journal.pone.0318087 (PMC11790111; doi:10.1371/journal.pone.0318087)
Supplement: Attachment — Submitted filename: Response to the reviewers.docx [file pone.0318087.s002.docx]

**Dear Reviewers,**

Thank you for the review of this paper. We appreciate the detailed feedback provided by the reviewers. We have carefully addressed each point raised, and the manuscript has been revised accordingly. Below is our point-by-point response:

# Reviewer #1:

1. **Is there any periodic temporal trend in the number of burn first aid cases in the hospital?**

*Response: Thank you for this insightful question. However, assessing temporal trends in burn first aid cases was not one of our study objectives. We interviewed a variety of participants, including current burn victims, caretakers, and visitors. Among the burn victims, some had sustained their injuries much earlier and had been on the hospital ward for an extended period, while others had only recently presented with burns. As a result, we did not collect specific data on when the burn injuries occurred, which would have been necessary to evaluate temporal trends.*

2. **Please make a necessary explanation on the formula for sample size calculation in your paper**

*Response: The formula used for sample size calculation has now been clarified in the "Study population and sample size" section. We included explanations of the symbols and assumptions (e.g., Z-score, π) as suggested.*

*The sample size was calculated using the formula: n = (z^2 * π * (1 - π)) / p^2, where we assumed a population proportion (π) of 50% for adequate burn first aid knowledge, a 95% confidence interval (z), and a margin of error (p) of 5%. This resulted in a required sample size of 384 participants. To account for a possible 5% non-response rate, we increased the sample to 404 participants, who were recruited through convenience sampling. The study included burn patients, their caretakers, and hospital visitors aged 12 years and above from the emergency and burn wards of KNRH, all of whom were invited to participate voluntarily.*

*In our study, we calculated the sample size using the following formula for estimating proportions:*

*n = (z² * π * (1-π)) / p², where:*

*n is the required sample size,*

*z is the z-score corresponding to the desired confidence level (for a 95% confidence interval, z = 1.96),*

*π represents the estimated proportion of the population that has adequate knowledge of burn first aid (BFA), which we assumed to be 50% as a conservative estimate when no prior data is available,*

*p is the margin of error, set at 5% (0.05).*

*This calculation yielded a minimum sample size of 384 participants. To account for potential non-response or incomplete data (estimated at 5%), we increased the sample size to 404 participants.*

*We used convenience sampling to enroll all burn patients, their caretakers, and visitors aged 12 years and above present in the emergency and burn wards of KNRH. While convenience sampling was practical for this setting, we acknowledge that it may limit the generalizability of the results, and this is addressed in the study’s limitations.*

*Line 128-144*

3. **Provide information on the main causes of burn injuries in the dataset.**

*Response:* We have added a new subsection in the results addressing the common causes of burn injuries, as derived from our dataset with a table

We sought to determine what current burn victims cause of burns. We also added a graph to summarise the cause as advised. Thank you for the suggestion again.

*Line 203-208*

4. **In this research, the logistic regression model was performed on a imbalanced data with 68 burn victims(16.8%). Did you consider using a weighted logistic regression model to address the issue of imbalanced data?**

**Response** *Thank you for your valuable insight regarding the imbalanced data in our study and the need for a weighte logistic regression model*

*Our population comprised current burn victims, caretakers, and visitors. For the multivariate logistic regression regarding factors associated with burn first aid knowledge, we considered all 404 participants. However, for the multivariate logistic regression on water lavage usage, we only included current burn victims and those who had experienced burns within the past year, resulting in a total of 94 participants in the model. This decision was made because many caretakers and visitors had never experienced burns themselves, making them unsuitable for this particular analysis. We thus constructed the second model to determine factors associated with water lavage usage using 94 participants.*

# Reviewer #2:

1. **On page 1 of the manuscript, the results part in the abstract stated that the "Mean BFA knowledge score was 56% (SD 13.9)." Given that the total possible score for the questionnaire is 13, the reported SD of 13.9 is surprisingly high. Should this value be expressed as a percentage (i.e., 13.9%) to reflect the variability relative to the mean percentage score? The same statement was also found on page 5 right under Table 1.**

*Response: Thank you for your insightful comment. The reported standard deviation of 13.9 is indeed related to the mean percentage score of 56%. We initially considered presenting the scores as percentages because we defined adequate knowledge as 80%, based on Bloom's cutoff score. However, we recognize that this could lead to confusion for readers. To clarify, we have now included both the actual score and its standard deviation, alongside the percentage score. This should enhance the understanding of the variability in relation to the mean percentage score.*

*Line 39 and Line 211-213*

2. **On page 2 of the manuscript, in the third paragraph of the introduction, the authors discussed that LMICs experience higher morbidity and mortality from burn injuries compared to HICs. While this assertion underscores the critical need for disseminating BFA knowledge in LMICs, it appears to lack data backing. Could the authors provide data to substantiate this claim? The inclusion of such data comparison would greatly reinforce the argument for the importance of BFA education in LMICs.**

*Response: Thank you so much for this correction. We have appropriately added more data to support this claim and references from relevant studies to substantiate the higher morbidity and mortality rates from burn injuries in LMICs compared to HICs.*

*Line 67-68*

3. **On page 3 of the manuscript, in the "Study population and sample size" subsection of the Methods. The authors use several mathematical symbols whose meanings may not be immediately clear to all readers.To prevent any potential misinterpretation, it would be beneficial for the authors to provide explicit definitions for these symbols. For instance, could the authors clarify that z represents the Z-score corresponding to a 95% confidence interval, and that π denotes the assumed population proportion? Elaborating on these symbols will enhance the manuscript's clarity and accessibility.**

*Response: Thank you so much for this suggestion; definitions for z (Z-score) and π (population proportion) have been included to make this section clearer to readers.*

*We edited the* ***Study population and sample size appropriately to this;***

*The sample size was calculated using the formula: n = (z^2 * π * (1 - π)) / p^2, where we assumed a population proportion (π) of 50% for adequate burn first aid knowledge, a 95% confidence interval (z), and a margin of error (p) of 5%. This resulted in a required sample size of 384 participants. To account for a possible 5% non-response rate, we increased the sample to 404 participants, who were recruited through convenience sampling. The study included burn patients, their caretakers, and hospital visitors aged 12 years and above from the emergency and burn wards of KNRH, all of whom were invited to participate voluntarily Line 128-144*

4. **In the sample size calculation detailed under the "Study population and sample size" section, the authors have opted to use a π=50% for the potential largest sample size required. Given that the actual population proportion in this study is significantly lower than 50%, a brief explanation of using π=50% would be beneficial for understanding of broader readers.**

*Response: Thank you for raising this important point. We opted to use π=50% in our sample size calculation as it provides the most conservative estimate, ensuring the largest possible sample size. This is a standard approach when there is limited prior knowledge about the population proportion, as it maximizes the sample size needed to detect significant differences. Although the actual population proportion in our study turned out to be lower than 50%, using π=50% allowed us to ensure adequate statistical power and robustness of the study's findings. We have added this explanation to the manuscript for clarity.*

*Line 132-134*

5. **On page 7 of the manuscript, under Figure 1, the authors note that among those with inadequate BFA knowledge, a substantial proportion were from the central region (77.3%, n=17), female (68.2%, n=15), and aged between 19 to 35 years (59.1%, n=13). The statement might be misleading as these demographics (central region, female, and 19-35 years) appear to represent the majority of the study's sample population. Could the authors clarify whether the high percentages reported are indications of these groups' lack of first aid training rather than a reflective of the sample's composition?**

*Response: I have edited this section such that we only mention that there were no significant differences in the characteristics.*

*Line 235-241*

**6. Some minor comments are as follows.**

a). Figure 1 and 2 currently missing captions and labels. Could the authors provide captions and labels to figures to enhance the reader's understanding of these visual elements?

b). In Table 2, row 5, there appears to be a typo: "pricking blisters" instead of "picking blisters."

c). In Table 2, row 8 and row 9, it should be "In case of" instead of "Incase of".

d). On page 3 of the manuscript, within the Methods section under "Data collection, sampling procedures and study measurements" subsection, the fourth row from the bottom, the authors mention several demographic factors (sex, age, education level, level of education, etc.). The terms "education level" and "level of education" are used redundantly. Could the authors consolidate these terms to eliminate repetition?

*Response: Captions and labels have been added to Figures 1 and 2. The typos identified in Table 2 and the redundancy in the demographic factors section have been corrected.*

*Thank you for this correction*

*Line 150, 223*

**Reviewer #3:**

**The study conducted in Uganda reveals a significant lack of burn first aid (BFA) knowledge and practice among the surveyed population, highlighting the urgent need for public education and improved access to accurate first aid information. However, there are several questions that need to be answered before considering the publication.

1) The introduction provides sufficient background information, but further discussion of the unique burn issues in Uganda could be added.**

*Response*

*Response: Thank you so much for this suggestion. We have edited the discussion which includes an overview of the specific challenges related to burn injuries in Uganda.*

Line 84-98

2**) The study design is clearly described, but the representativeness of the sample should be ensured in order to generalize to a wider Ugandan population.**

*Response:*

*Thank you for your valuable feedback regarding the representativeness of our sample. We recognize that there are limitations to generalizing our findings to the broader Ugandan population. Given our data collection was conducted over a period of five months, we aimed to capture a diverse range of participants, including burn victims, caretakers, and hospital visitors. The inclusion of visitors likely reflects the demographic diversity of the Ugandan population. Given that our center is the top referral unit, patients come from various regions of Uganda, as many individuals seek care at our tertiary center, which serves as a referral unit for burn cases across the country.*

*However, we understand that our sample may not fully represent all segments of the population affected by burns. This limitation has been highlighted in the manuscript's discussion section.*

*To further improve representativeness, we suggest that future studies consider employing stratified sampling methods or including additional centers to capture a broader demographic spectrum. We appreciate your insightful comment, which has prompted us to clarify these considerations in our revised manuscript*.

Line 323-327

**3) The statistical methods used are appropriate, but a detailed explanation of model selection and assumptions is needed.**

*Reply;*

*For BFA knowledge, we applied both simple bivariable and multivariable logistic regression. Initially, all factors—including demographic variables and prior exposure to BFA care or training—were included in the bivariable analysis. However, variables with small sample sizes, such as caring for a burn victim, receiving burn information, and BFA training, were excluded from the multivariable model to maintain statistical power and model stability.*

*For the analysis of water lavage practices, we similarly included all variables in the initial bivariate analysis. In the final multivariable model, we excluded variables with very small counts, such as "region" and "whether participants received BFA training," to improve model fit by removing data with low frequency and null values*

*This has been capture on Lines 172 to 181*

**4) The discussion should elaborate on the implications of the results and compare them with existing literature.**

*Reply;*

*Thank you for this valuable feedback. We have carefully elaborated on the implications of our findings in the discussion. We have also made thorough comparisons with existing literature, emphasizing how our results align or differ from previous studies. Additionally, we have highlighted the need for targeted burn first aid (BFA) education campaigns both in hospital settings and broader communities, as these could play a crucial role in improving BFA knowledge and practices. Line 271-342*

**5) The manuscript should be carefully checked for grammatical errors, spelling mistakes or unclear presentation.**

***Response***

*Thank you for your valuable feedback regarding the clarity and presentation of our manuscript. We acknowledge the importance of ensuring that our work is free from grammatical errors and spelling mistakes, as these can affect readability and comprehension.*

*In response to your comment, we have conducted a thorough review of the manuscript to identify and correct any such issues. We also sought assistance from other editors to enhance the overall clarity and coherence of the text. We believe these revisions will improve the quality of our presentation and make our findings more clear to the future readers.*

**Reviewer #4:**

**In this study, the authors assessed knowledge and practices of Prehospital Burn First Aid (BFA) among 404 participants, including burn victims, caregivers, and visitors. Findings revealed significant deficiencies: 83.9% had never received BFA information, 97.0% lacked first aid training, and only 5.4% demonstrated adequate BFA knowledge. Water lavage, a crucial BFA component, was used by only 27.7% of burn victims. No demographic factors showed significant associations with BFA knowledge or water lavage usage. This study indicated that addressing these gaps through community-based initiatives is crucial for improving burn care in Uganda.

In this study, the authors conducted a statistical description highlighting deficiencies in BFA knowledge and training. However, the study does not include further analysis to offer new insights into mitigating the risk of burns, indicating a lack of novel findings.**

**Reply**

*We appreciate your feedback regarding the analysis of BFA knowledge and training. In our study, we thoroughly examined an extensive range of variables to determine their associations burn first aid knowledge and water lavage practices. Although none of these variables were found to be significantly associated with the outcomes, this analysis contributes valuable insights into the current state of BFA knowledge and water lavage practices in a low-income setting.*

*Our findings underscore the concerning levels of knowledge and limited training, and misconceptions related to BFA among participants, highlighting critical gaps that can be addressed through targeted educational interventions. By documenting these deficiencies, our study adds new knowledge to the field and sets the stage for future efforts aimed at improving burn care in similar contexts.*
