# Supplementary material for: Exploring burn first aid knowledge and water lavage practices in Uganda: A cross-sectional study
Source: PLoS One. 2025 Feb 3;20(2):e0318087. doi: 10.1371/journal.pone.0318087 (PMC11790111; doi:10.1371/journal.pone.0318087)
Supplement: Attachment — Submitted filename: Response to Reviewers_.docx [file pone.0318087.s003.docx]

Response to Reviewers

Title: **Burn First Aid Knowledge and Water Lavage Practices Among Victims of Burn Injuries, Their Caregivers, and Visitors at a Tertiary Hospital in Uganda**

Dear Dr. Xiaosheng Tan and Reviewers,

Thank you so much for the opportunity to revise and resubmit our manuscript. We appreciate the feedback provided by the reviewers and have carefully addressed each comment to improve the manuscript's clarity, rigor, and impact. Below, we provide detailed responses to each point raised by the reviewers, along with a summary of the revisions made to the manuscript.

Reviewer #1

Comment 1: Please improve the quality of the paper writing.

Response: We have carefully revised the manuscript to enhance its readability and ensure that the language is clear, concise, and professional. Specifically, we focused on removing typing errors, improving sentence structure, eliminating redundant phrases, and ensuring consistency in terminology.

Comment 2: This is a cross-sectional study. Why don't you use the title "Exploring Burn First Aid Knowledge and Water Lavage Practices in Uganda: A Cross-Sectional Study"?

Response: We agree with the suggestion and have revised the title to:

"Exploring Burn First Aid Knowledge and Water Lavage Practices in Uganda: A Cross-Sectional Study."

Reviewer #4

Comment 1: The topic addressed in the manuscript does not make a substantial contribution to the field. The study relies on a questionnaire rather than experimental or other quantitative methods, which limits the depth and robustness of the findings.

Response:

Thank you for this critique. While the study uses a questionnaire-based design, it addresses a critical gap in understanding burn first aid knowledge and practices in Uganda, a region with limited data on community and caregiver knowledge and practices related to burn first aid. This information is essential for informing public health interventions and policies aimed at improving burn outcomes. Moreover, the findings from this study have directly contributed to a Quality Improvement (QI) project at the hospital, where we are working to distribute educational materials, such as posters and booklets, to burn victims and their families. These materials will focus on the correct water lavage practices following burns. Additionally, we plan to initiate community outreach programs and radio sensitizations to further raise awareness about burn prevention and burn furst aid. We have emphasized the significance and practical relevance of this work in the manuscript.

Comment 2: The sample size used in the study is insufficient to support robust statistical analysis or draw reliable conclusions.

Response:

We acknowledge the concerns regarding the sample size.. We have clarified the sample size calculation and its justification in the Methods section. Additionally, we have included a discussion of the study's limitations, including sample size, and their potential impact on the findings in the revised

Other Revisions

Language Improvements: Edited for clarity, conciseness, and readability throughout the manuscript.

We have thoroughly reviewed and updated the reference list to ensure all citations are accurate and complete. All references are appropriately active and none of the papers has been retracted.

We have uploaded the following documents as part of our resubmission:

Revised Manuscript with Track Changes (labeled "Revised Manuscript with Track Changes").

Clean Revised Manuscript (labeled "Manuscript").

This Rebuttal Letter (labeled "Response to Reviewers").

We hope the revisions adequately address the reviewers' concerns and improve the manuscript's overall quality. We look forward to your feedback and are happy to provide further clarifications if needed.

Sincerely,

Brian Kasagga

On behalf of all co-authors
